# Antimicrobial activity and comparative metabolomic analysis of *Priestia megaterium* strains derived from potato and dendrobium

**DOI:** 10.1038/s41598-023-32337-6

**Published:** 2023-03-31

**Authors:** Jia-Meng Liu, Yan-Tian Liang, Shan-Shan Wang, Nuo Jin, Jing Sun, Cong Lu, Yu-Feng Sun, Shu-Ying Li, Bei Fan, Feng-Zhong Wang

**Affiliations:** 1 Key Laboratory of Agro-Products Quality and Safety Control in Storage and Transport Process, Ministry of Agriculture and Rural Affairs, Beijing, China/Institute of Food Science and Technology, Chinese Academy of Agricultural Sciences, Beijing, China; 2 College of Pharmacy, Hunan University of Traditional Chinese Medicine, Hunan, China

**Keywords:** Microbiology, Bacteria, Environmental microbiology

## Abstract

The growth of endophytic bacteria is influenced by the host plants and their secondary metabolites and activities. In this study, *P. megaterium* P-NA14 and *P. megaterium* D-HT207 were isolated from potato tuber and dendrobium stem respectively. They were both identified as *Priestia megaterium*. The antimicrobial activities and metabolites of both strains were explored. For antimicrobial activities, results showed that *P. megaterium* P-NA14 exhibited a stronger inhibition effect on the pathogen of dendrobium, while *P. megaterium* D-HT207 exhibited a stronger inhibition effect on the pathogen of potato. The supernatant of *P. megaterium* P-NA14 showed an inhibition effect only on *Staphylococcus aureus*, while the sediment of *P. megaterium* D-HT207 showed an inhibition effect only on *Escherichia coli*. For metabolomic analysis, the content of *L*-phenylalanine in *P. megaterium* P-NA14 was higher than that of *P. megaterium* D-HT207, and several key downstream metabolites of *L*-phenylalanine were associated with inhibition of *S. aureus* including tyrosine, capsaicin, etc. Therefore, we speculated that the different antimicrobial activities between *P. megaterium* P-NA14 and *P. megaterium* D-HT207 were possibly related to the content of *L*-phenylalanine and its metabolites. This study preliminarily explored why the same strains isolated from different hosts exhibit different activities from the perspective of metabolomics.

## Introduction

Endophytes are microorganisms that exist inside plants and are not harmful to their hosts. In recent years, endophytes have attracted extensive attention, since they play an important role in enhancing plant growth and maintaining plant health^1,2^. Bacterial endophytes can provide various benefits for host plants, especially in promoting growth and resisting pathogens^3^ or inducing the innate immune system of plants^4^. Several research findings have revealed that the endophytes can promote plant growth by fixing nitrogen, making nutrient acquisition from soil, producing antimicrobial metabolites and modulating phytohormones status of plants^3,5^. In addition, endophytes can protect plants against biotic and abiotic stresses^6–8^. The development of new products such as endophyte-based microbial formulations and inducers shows great potential to improve plant resistance and provide abundant sources of important secondary metabolites.

In recent years, endophytic bacterial diversity of various plant species has been studied. The most frequently isolated bacterial genera include *Bacillus*, *Stenotrophomonas*, *Burkholderia* and *Pseudomonas*, while *Bacillus* is one of the main genera^9–11^. The taxonomy of *Bacillus* has recently been revisited through comprehensive phylogenomic and comparative genomic approaches^12^. *Bacillus megaterium* was renamed as *Priestia megaterium*, which is a new separate genus from *Bacillus*. According to the reports, *P. megaterium* is considered as a potential biological control agent, which has antimicrobial activities and a variety of control effects on plant diseases^13–15^. *P. megaterium* can be isolated from various plants, such as alfalfa, black pepper, carrot, clover, cotton, cucumber, potato, wheat, ginseng, dendrobium, polygonatum sibiricum^5,16,17^. Three different mechanisms to promote the plant growth by *P. megaterium* have been described in previous studies. Firstly, *P. megaterium* can secrete organic acids that providing the foundation of phosphate solubilization^18–20^. Secondly, *P. megaterium* can be responsible for concentration changes of phytohormones and other regulators of plant growth^21–23^. Thirdly, *P. megaterium* can also act as a biopesticide or biocontrol agent^24,25^.

For instance, *P. megaterium* strain B388 was isolated from rhizosphere soil of pine at Jageshwar, District Almora, Uttarakhand in India. *P. megaterium* B388 inhibited the growth of phytopathogens such as *Alternaria alternata* and *Fusarium oxysporum* by producing the spreadable and volatile compounds^26^. Kong et.al demonstrated that the *P. megaterium* extracted from marine bacterium could be used as a biocontrol agent in 2010^27^. The result of this study demonstrated that the *P. megaterium* from the Yellow Sea of East China exhibited significant activity in reducing infections caused by *Aspergillus flavus* on peanut seeds for the first time. At the same time, Xie et al. found that β-sitosterol, behenic acid, and phenylacetic acid extracted from *P. megaterium* L2 exhibited ideal antimicrobial activity against three gram-negative plant pathogen indicator bacteria as followed: *Agrobacterium tumefaciens* T-37, *Erwinia carotovora* EC-1 and *Ralstonia solanacearum* RS-2^28^.

In this research, two *P. megaterium* strains from different hosts (potato and dendrobium) were used as the studied strains. Both strains were evaluated by 16S rDNA sequences analysis as well as genomic analysis. Meanwhile, the two strains were evaluated for antimicrobial activity against five phytopathogens and human pathogens, including *Pectobacterium atroseptica*, *Athelia rolfsii*, *Staphylococcus aureus* and *Escherichia coli*. To further explore the different metabolites and metabolic pathways of these two strains, the extracts were submitted to liquid chromatography-mass (LC–MS) for nontargeted metabolomics. The reasons of why the same species in different hosts showed the different antimicrobial activities were preliminarily explored from perspective of metabolomic.

## Materials and methods

### Identification of endophytic bacteria

#### 16S rDNA molecular identification and phylogenetic tree analysis

Two endophytic strains of P-NA14 and D-HT207 were isolated from potato and dendrobium samples according to the method of our previous studies, respectively^29,30^. Extraction of endophytic bacteria DNA and sequencing was according to the method described by Wang et al*.* and Zhou et al.^29,31^. Related sequences were aligned and the phylogenetic tree was pictured using the maximum likelihood method with MEGA 7.0 software.

All plant materials used in this study comply with national guidelines. In addition, experimental research on endophytic bacteria was strictly comply with the IUCN Policy Statement on Research Involving Species at Risk of Extinction and the Convention on the Trade in Endangered Species of Wild Fauna and Flora.

#### Biochemical and physiological characterization

Based on Bergey’s Manual of Systematic Bacteriology, the identification of *P. megaterium* P-NA14 and *P. megaterium* D-HT207 was performed on morphological and biochemical characterization^32^. *P. megaterium* P-NA14 and *P. megaterium* D-HT207 were characterized based on the following biochemical tests: Hydrogen sulphide production test, Gelatin Liquefaction test, Methyl red test, Starch hydrolysis test, Urease test, Catalase test, Indole production test, Oxidase test, Voges-Proskauer test, Nitrate reduction test^33,34^.

#### High-throughput genome sequencing and genomic similarity analysis

DNA extracted from the *P. megaterium* P-NA14 and *P. megaterium* D-HT207 were subjected to whole genome sequencing by HiSeq × 10 platform (Illumina) following the supplier’s protocol (Illumina, UK), respectively.

The evaluation of similarity between the Coding sequence (CDS) of *P. megaterium* P-NA14 and *P. megaterium* D-HT207 was performed by BLAST. The protein sequences obtained were selected and aligned by GTDB-Tk v0.3.2^35^. Values of average nucleotide identity (ANI) and average amino acid identity (AAI) were calculated using the program JSpecies, version 1.2.1^36^. The sequences and annotations of other strains analyzed in this study were obtained from the NCBI database (http://www.ncbi.nlm.nih.gov).

### Antimicrobial activity

#### Endophytic bacteria fermentation and extraction

The Fermentation conditions of *P. megaterium* P-NA14 and *P. megaterium* D-HT207 were exactly the same. Single colonies of those two strains were injected into 50 mL liquid YIM38 medium^29^ and cultured at 200 rpm at 28 °C until bacteria reached 10^7^ mL^−1^ CFU (colony-forming unit), respectively. Then 20μL seed liquid was injected into 150 mL liquid YIM38 medium and cultured at the same conditions for 48 h, respectively. The culture liquid was centrifuged at 9500 rpm at 4 °C for 20 min. Then the supernatant and deposit were collected respectively.

The supernatant was mixed with an equal volume of ethyl acetate and collected by a separating funnel, then dried by rotary evaporator (RE-52AA; YARONG, China) at 38 °C. The sediment mixed with 15 mL of acetone for 12 h was filtered through a 0.22 μm nylon membrane filter and dried by a rotary evaporator. The extracts of supernatant and sediment were dissolved in 1 mL methanol and stored at − 20 °C prepared for experiments, respectively.

#### Antimicrobial activity screening

The antimicrobial activities of *P. megaterium* P-NA14 and *P. megaterium* D-HT207 were evaluated by five indicator strains as listed in Supplementary Table S1. *Pectobacterium atroseptica* was obtained from the Agricultural Culture Collection of China (ACCC). *Athelia rolfsii* was obtained from the Sanming Academy of Agricultural Sciences, Fujian Province, China. *Staphylococcus aureus* and *Escherichia coli* were deposited in Institute of Medicinal Biotechnology, Chinese Academy of Medical Sciences & Peking Union Medical College. Antimicrobial activities were tested by Kirby-Bauer test^37^. Twenty microliters of each sample were loaded on sterilized filter paper with a diameter of 6 mm. An equal amount of methanol was used as the negative control. Samples were cultured at 28 °C for 20 h. Antimicrobial activities were evaluated by measuring the diameter of the inhibition zones using an electronic digital caliper (0–150 mm).

### Metabolomics comparative analysis

#### Metabolite extraction

The supernatant was dried and redissolved in 100µL resolution (acetonitrile: water = 1:1, v/v), then it was transferred to sample bottles for LC–MS/MS analysis after centrifugation at 13,000 rpm at 4 °C for 15 min. The samples of *P. megaterium* P-NA14 were named A14 group, and samples of *P. megaterium* D-HT207 were named A207 group during metabolomics analysis. Both samples were set as 6 parallel samples.

#### UPLC–TOF/MS analysis

Chromatographic separation of the metabolites was performed on ExionLC AD system (AB Sciex, USA) equipped with an ACQUITY UPLC BEH C18 column (100 mm × 2.1 mm i.d., 1.7 µm; Waters, Milford, USA). The UPLC system was coupled to a quadrupole-time-of-flight mass spectrometer (Triple TOFTM5600+, AB Sciex, USA). The optimal conditions were set as listed (Supplementary Tables S2, S3). Mobile phase A consisted of 0.1% formic acid in water, and mobile phase B consisted of 0.1% formic acid in acetonitrile: isopropanol (1:1, v/v). During the period of analysis, the rest samples were stored at 4 °C.

### Data analysis

After UPLC-TOF/MS analysis, the raw data were imported into the Progenesis QI 2.3 (Waters Corporation, Milford, USA). The extracted data included the retention time (RT), mass-to-charge ratio (m/z) values, and peak intensity. Mass spectra of these metabolic features were identified by using the accurate mass fragments spectra and isotope ratio difference searching from reliable biochemical databases such as Metlin database (https://metlin.scripps.edu/). The internal standard was used for data QC (reproducibility), deleting variables with Relative Standard Deviation (RSD) > 30% in the QC samples.

### Multivariate statistical and differential metabolites analysis

All multivariate statistical data analysis was performed using ropls (Version 1.6.2) from Bioconductor on Majorbio Cloud Platform (https://cloud.majorbio.com). Unsupervised principal component analysis (PCA), partial least squares discriminant analyses (PLS-DA) and orthogonal partial least squares discriminant analyses (OPLS-DA) were performed to distinguish the overall differences in metabolic profiles between groups and to find differential metabolites between groups. Variable importance in the projection (VIP) and fold change (FC) were calculated in the OPLS-DA model. *P* values were estimated with paired Student’s test on Single dimensional statistical analysis. Statistically significant among groups were selected with VIP > 1, FC > 2(or < 0.5), and *p* values < 0.05.

Differential metabolites among two groups were summarized and mapped into their biochemical pathways through metabolic enrichment and pathway analysis based on the KEGG database (http://www.genome.jp/kegg/). These metabolites can be classified according to the pathways they were involved in or the functions they performed. Scipy.stats (Python packages) (https://docs.scipy.org/doc/scipy/) was employed to identify statistically significantly enriched pathways using Fisher’s exact test.

## Results

### Identification of endophytic bacteria

#### 16S rDNA molecular identification and phylogenetic tree analysis

**Figure 1:**
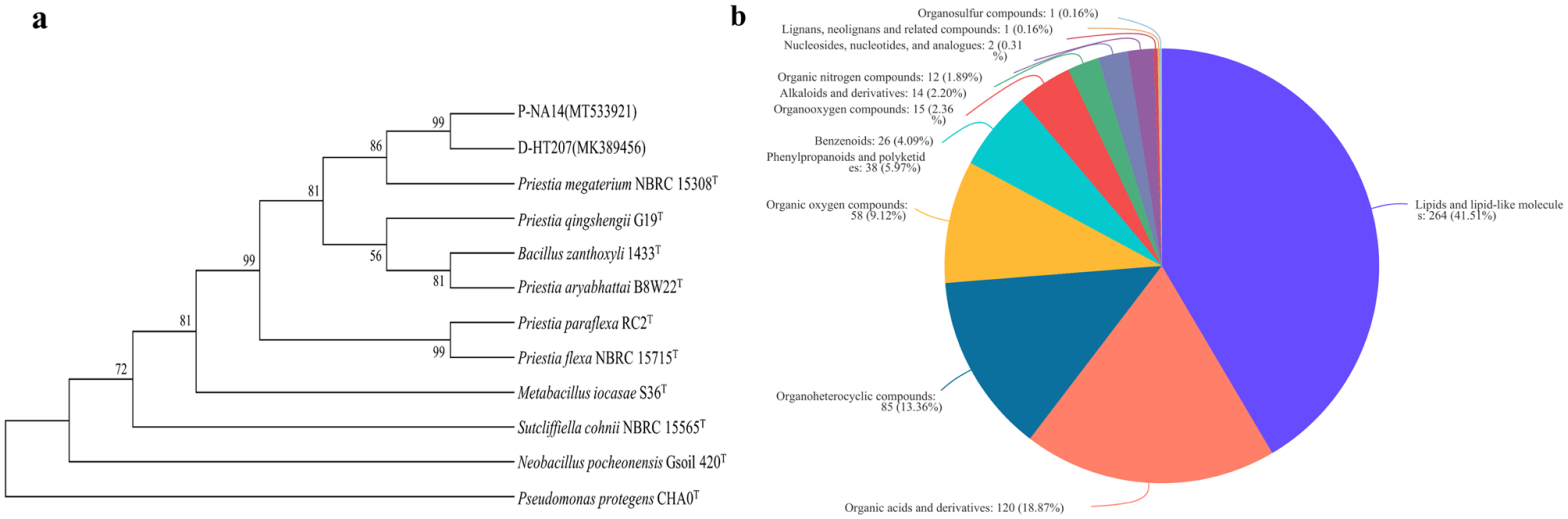
(**a**) The phylogenetic tree of *P. megaterium* P-NA14 and *P. megaterium* D-HT207 was identified in this study. The identification showed that *P. megaterium* P-NA14 and *P. megaterium* D-HT207 belonged to the same species. (**b**) Classification of the identified metabolites annotated using the Metlin database.

The 16S rDNA gene sequences of both strains were submitted to the NCBI GenBank to get the Accession Numbers. *P. megaterium* P-NA14 displayed 99.31% similarity with *P. megaterium* (Accession No. MT533921). *P. megaterium* D-HT207 displayed 99.12% similarity with *P. megaterium* (Accession No. MK389456). A phylogenetic tree was established for *P. megaterium* P-NA14 and *P. megaterium* D-HT207 to demonstrate their evolutionary relationship. The identification showed that *P. megaterium* P-NA14 and *P. megaterium* D-HT207 belonged to *P. megaterium* (Fig. 1a).

#### Biochemical and physiological characterization

The results of the physiological and biochemical reactions of *P. megaterium* P-NA14 and *P. megaterium* D-HT207 turned up to be exactly the same although they were isolated from different hosts (Supplementary Table S4).

#### High-throughput genome sequencing and genomic similarity analysis

The complete genome of *P. megaterium* P-NA14 has a total size of 4 929 750 bp assembled in one scaffold, with a 37.84% G + C content. The complete genome of *P. megaterium* D-HT207 has a total size of 5 108 847 bp assembled in one scaffold, with a 38.15% G + C content. The genomes from *P. megaterium* P-NA14 and *P. megaterium* D-HT207 were deposited in the NCBI GenBank database, under the accession number CP109761-CP109767 and CP109859-CP109862, respectively. Supplementary Table S5 demonstrated the comparison between the genome sequence of *P. megaterium* P-NA14 and *P. megaterium* D-HT207.

The phylogenomic analysis based on the concatenated alignment of 120 orthologous proteins showed that the *P. megaterium* P-NA14 and *P. megaterium* D-HT207 were identified as *P. megaterium* (Supplementary Table S6). ANI and AAI represent the average nucleotide and the amino acid identity, respectively, of all orthologous genes shared between any two genomes and offer robust resolution between strains of the same or closely related species^38,39^. The values of ANI and AAI between *P. megaterium* P-NA14 and *P. megaterium* D-HT207 genomes were 97.34% and 96.7% above the 96% threshold, respectively (Table 1). Furthermore, the ANI and AAI values indicated that *P. megaterium* P-NA14 and *P. megaterium* D-HT207 had the closest evolutionary relationship with *P. megaterium* NBRC 15308. This result indicated that genomes of *P. megaterium* P-NA14 and *P. megaterium* D-HT207 belonged to the same species.

**Table 1:** ANI and AAI values (%) between two strains and type strains of phylogenetically related species.

Genome A	Genome B	Mean ANI (%)	Mean AAI (%)
P-NA14	D-HT207	96.7	97.34
P-NA14	***P****. ****megaterium* NBRC 15308**	**96.8**	**97.24**
P-NA14	*B. aryabhattai* B8W22	95.4	96.58
P-NA14	*B. flexus* NBRC 15715	75.2	73.86
D-HT207	P-NA14	96.7	97.34
D-HT207	***P****. ****megaterium* NBRC 15308**	**96.5**	**97.18**
D-HT207	*B. aryabhattai* B8W22	95.6	96.66
D-HT207	*B. flexus* NBRC 15715	75.4	74.47

The highest ANI and AAI values between two strains and type strains of phylogenetically related species are in bold text.

### Antimicrobial activity

The antimicrobial activities of *P. megaterium* P-NA14 and *P. megaterium* D-HT207 against five different pathogens were evaluated. The results showed inhibition diameters of *P. megaterium* P-NA14 and *P. megaterium* D-HT207 (Table 2). *P. megaterium* P-NA14 and *P. megaterium* D-HT207 showed antimicrobial activity on plant pathogens but showed different activity on *S. aureus* and *E. coli*. For *P. atroseptica*, *P. megaterium* D-HT207 showed higher activity than *P. megaterium* P-NA14, and the supernatant of *P. megaterium* D-HT207 possessed the strongest antimicrobial activity. For *A. rolfsii*, *P. megaterium* P-NA14 showed higher activity than *P. megaterium* D-HT207, and the supernatant of *P. megaterium* P-NA14 possessed the strongest antimicrobial activity. The supernatant of *P. megaterium* P-NA14 showed inhibition against *S. aureus* ATCC 29213, while the sediment of *P. megaterium* D-HT207 showed inhibition against *E. coli*. *P. megaterium* P-NA14 and *P. megaterium* D-HT207 showed no inhibition against *S.aureus* ATCC 33591.

**Table 2:** Inhibition effect of fermentation broth of strain P-NA14 and strain D-HT207 on the different pathogens.

Strain	Active fraction	*P. atroseptica* (mm)	*A. rolfsii* (mm)	*S. Aureus* ATCC 29213 (mm)	*S. Aureus* ATCC 33591 (mm)	*E. coli* (mm)
P-NA14	Supernatant	10.43 ± 0.19	12.88 ± 0.26	11.23 ± 0.38	–	–
	Sediment	13.53 ± 1.11	8.06 ± 0.39	–	–	–
D-HT207	Supernatant	14.19 ± 1.62	10.23 ± 0.30	–	–	–
	Sediment	12.14 ± 0.52	8.95 ± 0.34	–	–	10.78 ± 0.29

### Metabolomics comparative analysis

To evaluate the metabolite changes of *P. megaterium* P-NA14 and *P. megaterium* D-HT207, the metabolic compounds of each sample were analyzed using an untargeted metabolomics approach. The results indicated that 4363 and 4881 metabolites were detected by the POS (positive) and NEG (negative) models, respectively. According to annotation in Metlin database, 437 compounds in POS and 389 compounds in NEG were defined as common compounds for A14 group and A207 group. After data preprocessing, 636 metabolites obtained from level-two identification were annotated to the Metlin database, which were matched and classified into 12 superclasses, including 264 lipids and lipid-like molecules; 120 organic acids; 85 organoheterocyclic compounds; 58 organic oxygen compounds; 38 phenylpropanoids and polyketides; 26 benzenoids; 15 organooxygen compounds; 14 alkaloids and derivatives; 12 organic nitrogen compounds; 2 nucleosides, nucleotides and analogues; 1 lignans, neolignans and related compounds and 1 organosulfur compounds (Fig. 1b).

#### Multivariate statistical analysis

##### Principal component analysis (PCA)

To get a preliminary understanding of the metabolites difference between A14 group and A207 group, the metabolic data matrix for A14 group and A207 group were analyzed using PCA plot. The PCA scores plot showed that the QC clustered tightly together (Supplementary Fig. S1), which indicated the QC repeatability was good and the stability of the analysis system was high.

**Figure 2:**
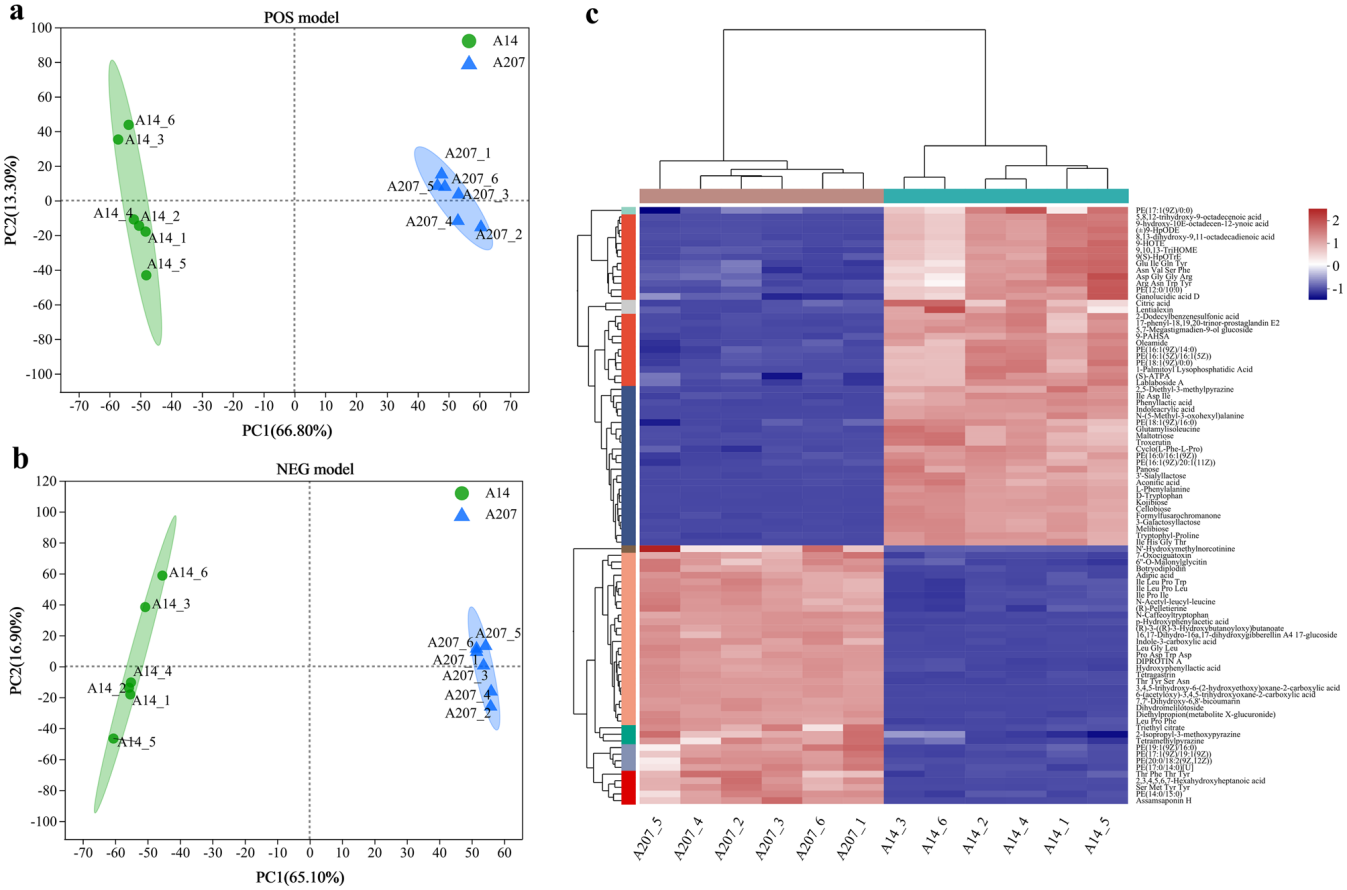
(**a**,**b**) PCA scores plot of A14 and A207 group in positive model and negative model. (**c**) Top 90 metabolites clustering heatmap of all samples using HCA, the horizontal direction were the samples, and the longitudinal direction were the identified metabolites.

The samples from the same group clustered, respectively. The A14 group was clustered together while A207 group was located on the opposite branch. Both the tested groups were well separated in the PCA plot, showing a remarkable variation in metabolism between two groups (Fig. 2a,b). The clustering heatmap also revealed significant variation in metabolites between A14 and A207 groups (Fig. 2c).

##### Partial least squares-discriminant analysis (PLS-DA)

**Figure 3:**
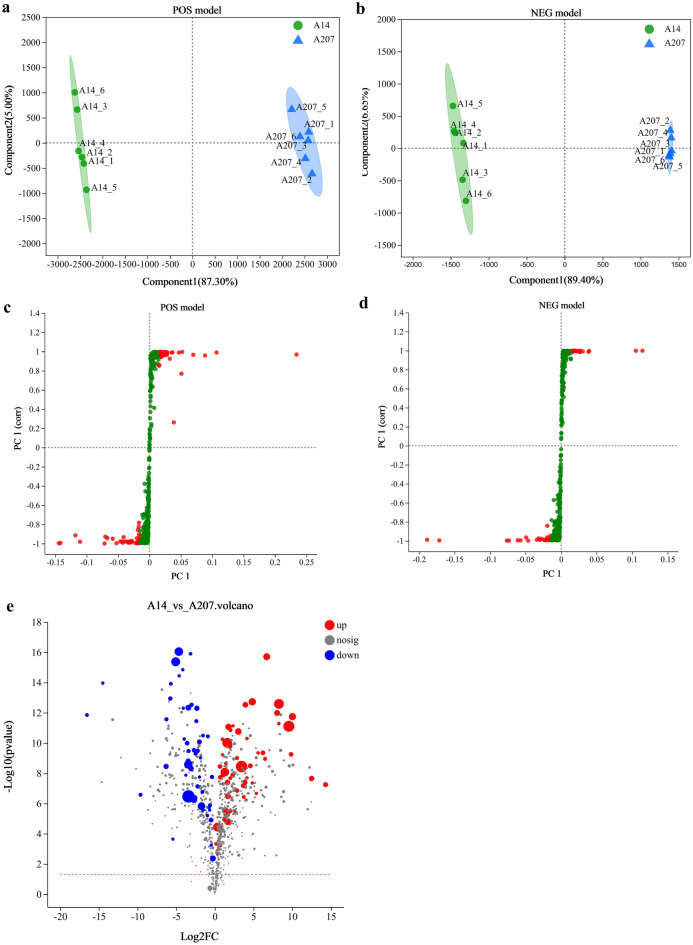
(**a**,**b**) PLS-DA scores plot derived from A14 and A207 each group in positive ion mode and negative ion mode. (**c**,**d**) S-plot based on OPLS-DA model (positive ion mode and negative ion mode). (**e**) Volcano map of differential metabolites between A14 group and A207 group. Each point in the figure represents a metabolite.

Similar to the PCA analysis, PLS-DA was used to maximize the slight difference between the samples, which was useful to detect differential metabolites. The six parallel samples of A14 group were distributed within the A14 group cluster, and six parallel samples of A207 group were within the A207 group cluster. In both positive and negative models, two clusters were clearly distinguished from the score plot, and the distribution trend was identical to the PCA results, indicating that there was a distinct difference between the A14 group and A207 group (Fig. 3a,b). A permutation test with 200 iterations was performed to evaluate the possible overfitting of the PLS-DA models. The R^2^ and Q^2^ were extremely close to 1, indicating the high accuracy in model fitting. The results indicated that the PLS-DA models had a great predictive capacity and might be performed for further metabolites variance analysis.

#### Differential metabolites analysis

OPLS-DA is a multivariate statistical analysis method with supervised pattern recognition, and can solve the problem that PCA is not sensitive to the variables with little correlation. The variables with VIP values larger than 1 were highlighted with red dots. These red dots were relevant variables to separate the two groups (Fig. 3c,d).

The statistics of differential metabolites between two endophytes were shown in Fig. 3e. There were 90 differential metabolites, which contained 51 up-regulated metabolites and 39 down-regulated metabolites (Supplementary Table S7), and 13 of the 90 differential metabolites were annotated to KEGG metabolic pathways, including cellobiose, melibiose, *L*-Phenylalanine, aconitic acid, maltotriose, 9(S)-HpOTrE, citric acid, phenyllactic acid, 9,10,13-TriHOME, adipic acid, 6''-O-Malonylglycitin, hydroxyphenyllactic acid, p-Hydroxyphenylacetic acid (Table 3).

**Table 3:** Significant differential metabolites between the comparison groups of A14 group and A207 group.

No.	Metabolite	Formula	FC (A14/A207)	VIP (OPLS-DA)	*P* value	RT	Mode	M/Z
1	Cellobiose	C_12_H_22_O_11_	1043.85	5.13	1.80E−12	0.72	Neg	387.11
2	Melibiose	C_12_H_22_O_11_	756.16	11.97	7.87E−12	0.65	Neg	377.09
3	*l*-Phenylalanine	C_9_H_11_NO_2_	310.95	9.56	2.62E−13	1.59	Pos	166.09
4	Aconitic acid	C_6_H_6_O_6_	90.02	1.58	1.12E−09	1.05	Neg	173.01
5	Maltotriose	C_18_H_32_O_16_	14.72	2.05	3.69E−08	0.65	Pos	527.16
6	9(S)-HpOTrE	C_18_H_30_O_4_	13.30	1.36	3.68E−07	5.87	Neg	309.21
7	Citric acid	C_6_H_8_O_7_	5.95	1.04	3.24E−06	0.68	Neg	191.02
8	Phenyllactic acid	C_9_H_10_O_3_	3.38	3.99	8.67E−12	3.17	Neg	165.06
9	9,10,13-TriHOME	C_18_H_34_O_5_	3.23	3.44	3.36E−07	4.98	Neg	329.23
10	Adipic acid	C_6_H_10_O_4_	0.35	1.37	3.24E−11	1.17	Neg	145.05
11	6''-O-Malonylglycitin	C_25_H_24_O_13_	0.25	1.13	1.79E−08	0.98	Pos	497.11
12	Hydroxyphenyllactic acid	C_9_H_10_O_4_	0.20	2.76	5.09E−13	2.39	Neg	181.05
13	P-Hydroxyphenylacetic acid	C_8_H_8_O_3_	0.11	1.32	1.27E−16	2.90	Neg	151.04

FC was the mean ratio between the A14 group and A207 group. Positive numbers represented that increasing in the A14 group compared with the A207 group, and negative numbers mean a decrease.

#### KEGG pathway analysis for differential metabolites

**Figure 4:**
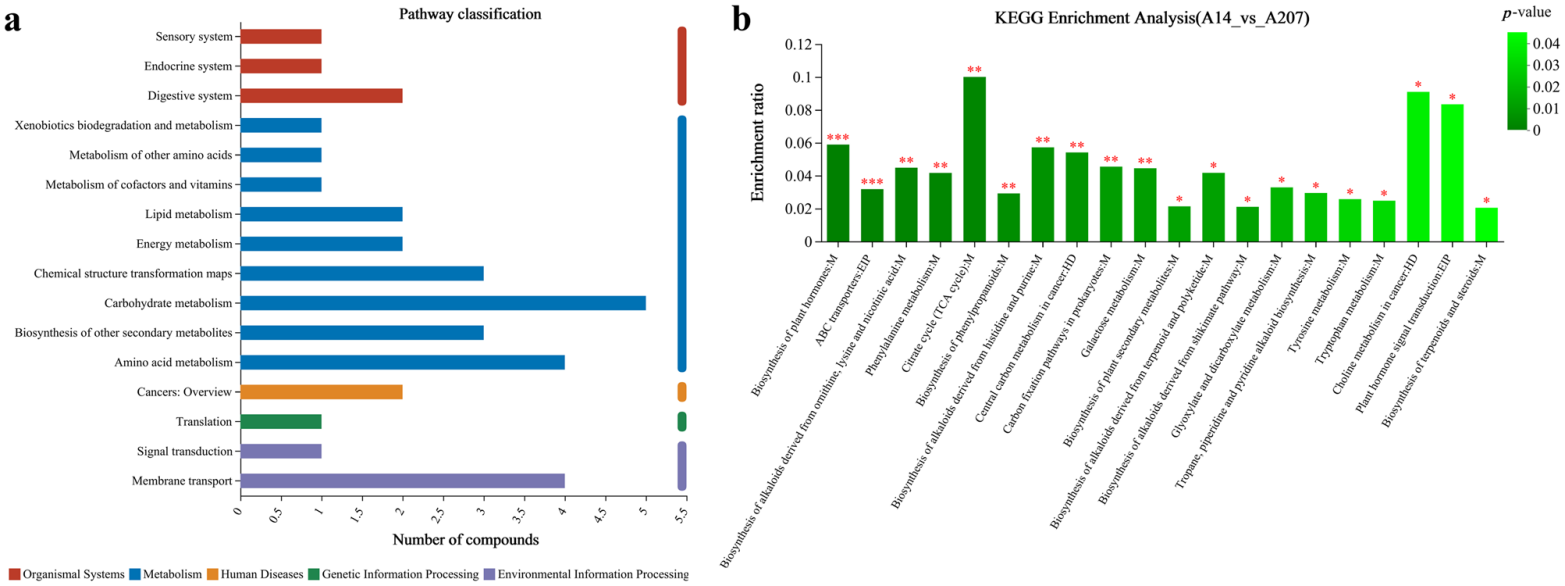
(**a**) Classification of differential metabolites between the A14 group and A207 group by KEGG. (**b**) The abscissa represents the pathway name. The ordinate represents the enrichment ratio, indicating the ratio of the metabolite number to the background number annotated to the pathway. The column color gradient indicates the significance of enrichment. *** indicates *p* < 0.001, ** indicates *p* < 0.01, * indicates *p* < 0.05.

KEGG enrichment analysis showed that 90 differential metabolites were annotated into 46 pathways and divided into 16 categories. The differential metabolites were mainly enriched in metabolism, organismal systems, environmental information processing, cellular processes (Fig. 4a). There were 20 metabolic pathways (*p* < 0.05) that showed higher enrichment ratio (Fig. 4b), including carbohydrate metabolism, energy metabolism, amino acid metabolism, biosynthesis of other secondary metabolites, chemical structure transformation maps, membrane transport, signal transduction, cancer: overview.

**Figure 5:**
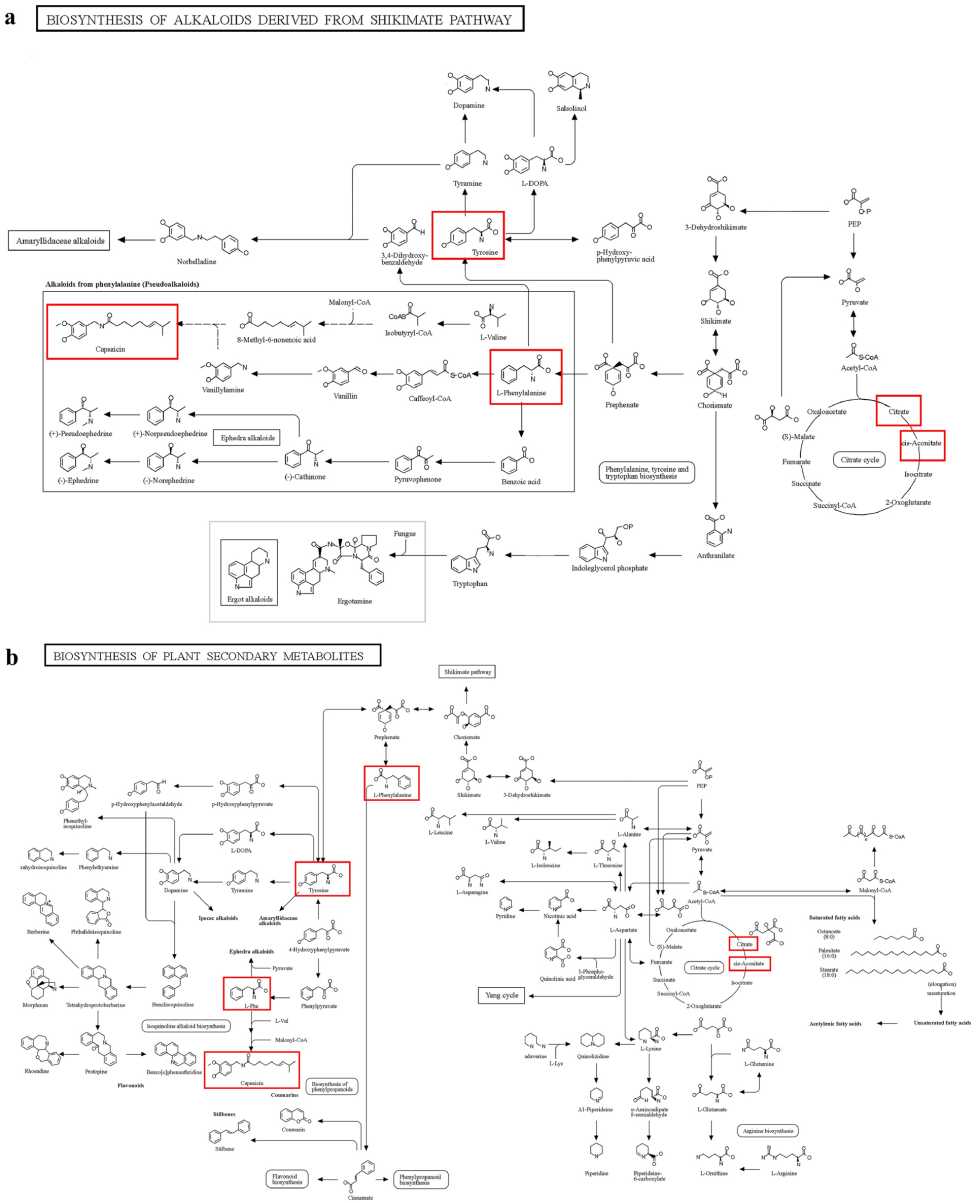
(**a**) Biosynthesis of plant secondary metabolites pathway. (**b**) Biosynthesis of alkaloids derived from shikimate pathway.

Two pathways showed the most significant differences (*p* < 0.001): Biosynthesis of plant hormones (map01070) and ABC transporters (map02010). In addition, the pathways that might explain the different antimicrobial activities of the two endophytes are as follows: biosynthesis of plant secondary metabolites (map01060); biosynthesis of alkaloids derived from shikimate pathway (map01063); biosynthesis of alkaloids derived from ornithine, lysine and nicotinic acid (map01064); biosynthesis of alkaloids derived from terpenoid and polyketide (map01066); biosynthesis of amino acids (map01230). The involved differential metabolites were aconitic acid, citric acid and *L*-Phenylalanine in this study. In those pathways, aconitic acid and citric acid were mainly involved in the tricarboxylic acid cycle. *L*-Phenylalanine might affect several downstream metabolites which could affect the antimicrobial activities of two endophytes: capsaicin, tyrosine, piperine, tomatine and geraniol. *L*-Phenylalanine was closer to related downstream metabolites in the biosynthesis of plant secondary metabolites pathway and biosynthesis of alkaloids derived from shikimate pathway (Fig. 5).

## Discussion

In recent years, endophytes have attracted extensive attention, since they play an important role in enhancing plant growth and maintaining plant health. Endophytic bacterial diversity of various plant species has been studied. *P. megaterium*, as one of the common endophytes, shows antimicrobial activities and a variety of control effects on plant diseases. In this research, antimicrobial activities against five pathogens of two *P. megaterium* strains have been explored. The possible reason for the different antimicrobial activities of the two *P. megaterium* strains was explored by analyzing metabolites with the metabolomic analysis and KEGG metabolic pathway annotation^40,41^. The present study provided a reference for the biological control of dendrobium phytopathogens and potato phytopathogens. In addition, the related metabolic pathways will provide a theoretical basis for the subsequent exploration of metabolites in *P. megaterium* that are isolated from others hosts.

### Antimicrobial activities of* P. megaterium* P-NA14 and *P. megaterium* D-HT207

In this research, the results of 16S rDNA gene sequencing and genomic analysis showed that *P. megaterium* P-NA14 and *P. megaterium* D-HT207 belonged to *P. megaterium*, and they had the closest evolutionary relationship with *P. megaterium* NBRC 15308. Previous studies have demonstrated that the genus *Bacillus* is well known for the natural production of secondary metabolites with antimicrobial activities and shows a very strong biocontrol potential, which works against phytopathogens such as *Burkholderia solanacearum* and *Fusarium oxysporum*^42,43^*.* Previous studies showed that the antimicrobial activity of *P. megaterium* D-HT207 against *A. rolfsii* was the highest among the endophytes isolated from dendrobium^29^, the antimicrobial activity of *P. megaterium* P-NA14 against *P. atroseptica* ACCC 19901 was the highest among the endophytes isolated from potato^30^. According to the results of our antibacterial test, *P. megaterium* P-NA14 showed a stronger inhibition effect on *A. rolfsii* than *P. megaterium* D-HT207. *P. megaterium* D-HT207 showed a stronger inhibition effect on *P. atroseptica* ACCC 19901 than *P. megaterium* P-NA14. Meanwhile, the antimicrobial activities of the two strains against *S. aureus* and *E. coli* were opposite. Interestingly, *P. megaterium* showed antimicrobial activity against both *S. aureus* and *E. coli* isolated from dairy waste, mango pulp waste and oral microflora^44,45^. At present, there is no research on the mechanism of different activities of strains isolated from different hosts^46,47^. It was speculated that in the long-term coevolution process of endophytic bacteria and host, the stimulation to their original host pathogens will gradually weaken, but the inhibitive activity to exogenous pathogens will be stronger.

### The differential metabolites

According to recent reports, the secondary metabolites of plant endophytes would be affected by different hosts. Dobrzanski et al*.* compared the adaptive responses of bacteria belonging to the same species that came from two different ecosystems (water and soil). The final balance of ROS produced was different between the two strains, but the related metabolic pathways was still not described yet^48^. A slight difference in the plant growth-promoting genes among the four *Bacillus cereus* strains isolated from different hosts (forest, haw, wheat, and alfalfa) was observed. The results of specific genes analysis showed that the differential genes were related to amino acid, carbohydrate and coenzyme transport and metabolism^49,50^. The differential metabolites of *P. megaterium* P-NA14 and *P. megaterium* D-HT207 were analyzed by UPLC-QTOF-MS, *L*-phenylalanine in *P. megaterium* P-NA14 is up-regulated compared to *P. megaterium* D-HT207. In plants, *L*-phenylalanine was found to be a precursor to a multitude of plant metabolites that are important in plant growth, nutrition, reproduction, resistance, and environmental response^51–53^. The increase of *L*-phenylalanine content may be the key reason for the differential changes of downstream metabolites.

### Related downstream metabolites of *L*-phenylalanine in the KEGG pathways

With the analysis of KEGG database^54,55^. *L*-phenylalanine might affect the synthesis of capsaicin, tyrosine, piperine, tomatine, and geraniol. Capsaicin and tyrosine were the closest downstream metabolites to *L*-phenylalanine in pathways (Fig. 5). Recent research showed that capsaicin had stronger antimicrobial activity on *S. aureus* than *E. coli*^56^*.* Capsaicin inhibited the NorA efflux pump of *S. aureus* and reduced the invasiveness of *S. aureus*^57^. Tyrosine affects the production of downstream glycopeptide antibiotics through the biosynthesis of vancomycin group antibiotics (map01055), which had an inhibitory effect on* S. aureus*. Piperine showed antimicrobial activity on *S. aureus* instead of *E. coli*. The tomatine and geraniol exhibited higher antimicrobial activity and MIC values of *S. aureus* than* E. coli*^3,58^. The antimicrobial activity of these five downstream metabolites against *S. aureus* is the same as that of *P. megaterium* P-NA14, indicating for the first time that the antimicrobial activity of *P. megaterium* against *S. aureus* may be related to the secondary metabolites. But the reason for the different inhibitory activity against *E. coli* still needs further investigation.

## Conclusion

The results confirmed in this study that the difference in antimicrobial activity between *P. megaterium* P-NA14 and *P. megaterium* D-HT207 was related to downstream metabolites affected by *L*-phenylalanine. Previous reports showed downstream metabolites affected by higher content of *L*-phenylalanine exhibited higher antimicrobial activity against *S. aureus* than* E. coli*. In summary, the antimicrobial activities of the same species isolated from different hosts turn out different. This study has proved that the secondary metabolites of plant endophytes were affected by the host, and it might be affected at the level of hormones, enzymes and genes. To clarify how the host affects endophytic bacterial products, it should be analyzed not only in metabolomics but also in target compounds and genomics levels.

## Supplementary Information

Supplementary Information 1.

Supplementary Information 2.

Supplementary Information 3.

Supplementary Information 4.

Supplementary Information 5.

## Data Availability

The authors confirm that the data supporting the findings of this study are available within the article and its supplementary materials.
