# Supplementary material for: Antimicrobial activity and comparative metabolomic analysis of Priestia megaterium strains derived from potato and dendrobium
Source: Sci Rep. 2023 Mar 31;13:5272. doi: 10.1038/s41598-023-32337-6 (PMC10066289; doi:10.1038/s41598-023-32337-6)
Supplement: Supplementary file 1 — Supplementary Information 1. [file 41598_2023_32337_MOESM1_ESM.pdf]

Antimicrobial activity and comparative metabolomic analysis of *Priestia megaterium* strains derived from potato and dendrobium

Jia-Meng Liu[†,1], Yan-Tian Liang[†,2], Shan-Shan Wang[1], Nuo Jin[1], Jing Sun[1], Cong Lu[1], Yu-Feng Sun[1], Shu-Ying Li[1], Bei Fan[1*], Feng-Zhong Wang[1*]

1 Key Laboratory of Agro-products Quality and Safety Control in Storage and Transport Process, Ministry of Agriculture and Rural Affairs, Beijing, China/Institute of Food Science and Technology, Chinese Academy of Agricultural Sciences, Beijing, China

2 College of Pharmacy, Hunan University of Traditional Chinese Medicine, Hunan, China

† These authors have contributed equally to this work and share first authorship.

*** Correspondence:**

Bei Fan: caasBFan@163.com

Feng-Zhong Wang: caasFZWang@163.com

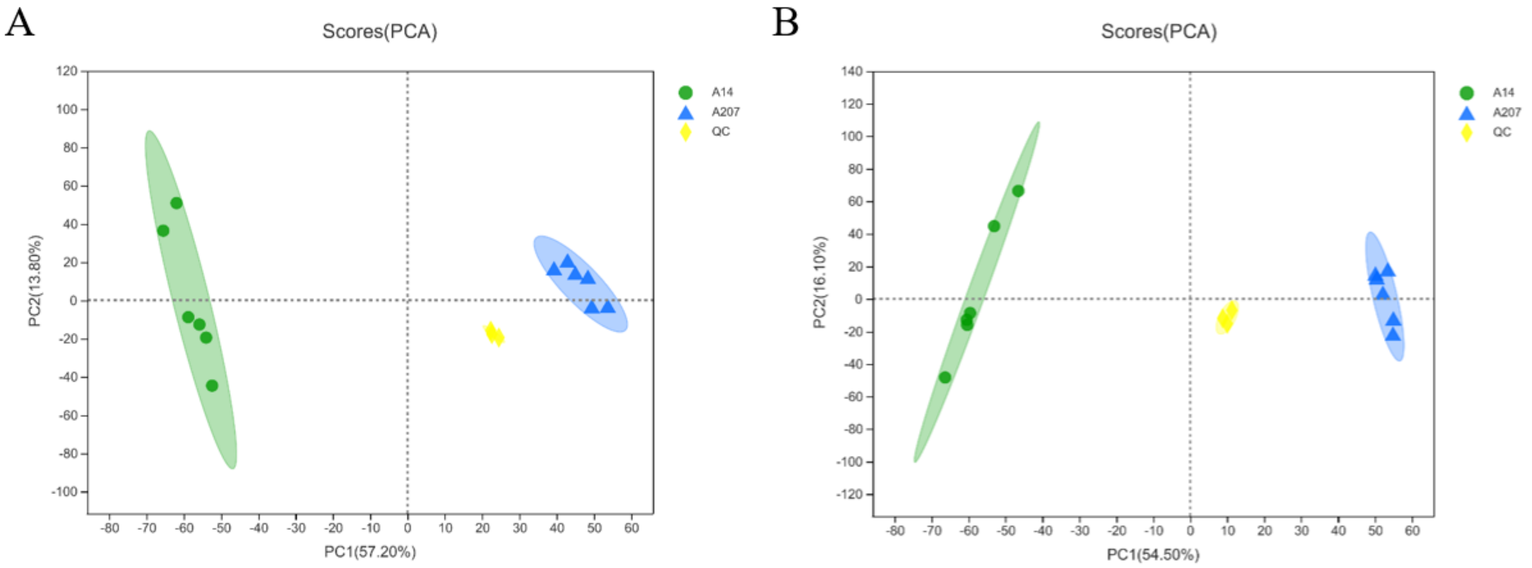

Figure S1: PCA scores plot of QC, A14 and A207 group in positive model(A) and negative model(B), indicating the QC repeatability was good and the stability of the analysis system was high.
