# Supplementary material for: Antimicrobial activity and comparative metabolomic analysis of Priestia megaterium strains derived from potato and dendrobium
Source: Sci Rep. 2023 Mar 31;13:5272. doi: 10.1038/s41598-023-32337-6 (PMC10066289; doi:10.1038/s41598-023-32337-6)
Supplement: Supplementary file 2 — Supplementary Information 2. [file 41598_2023_32337_MOESM2_ESM.pdf]

Antimicrobial activity and comparative metabolomic analysis of *Priestia megaterium* strains derived from potato and dendrobium

Jia-Meng Liu[†,1], Yan-Tian Liang[†,2], Shan-Shan Wang[1], Nuo Jin[1], Jing Sun[1], Cong Lu[1], Yu-Feng Sun[1], Shu-Ying Li[1], Bei Fan[1*], Feng-Zhong Wang[1*]

1 Key Laboratory of Agro-products Quality and Safety Control in Storage and Transport Process, Ministry of Agriculture and Rural Affairs, Beijing, China/Institute of Food Science and Technology, Chinese Academy of Agricultural Sciences, Beijing, China

2 College of Pharmacy, Hunan University of Traditional Chinese Medicine, Hunan, China

† These authors have contributed equally to this work and share first authorship.

*** Correspondence:**

Bei Fan: caasBFan@163.com

Feng-Zhong Wang: caasFZWang@163.com

Table S1. Indicator strains were used in this study.

**Strain**	**Type strain**	**Disease**
P. atroseptica	ACCC 19901	Potato blackleg disease
A. rolfsii	CBS719.83	Dendrobium southern blight
S. aureus drug-sensitive strain	ATCC 29213	food-borne pathogenic microorganism
S. aureus drug-resistant strain	ATCC 33591	
E. coli	ATCC 25922	

Table S2. Elution conditions of UPLC.

Time(min)	Flow rate(mL/min)	A (%)	B (%)	injection volume (μL)	column temperature (°C)
0	0.4	95	5	20	40
3	0.4	80	20		
9	0.4	5	95		
13.0	0.4	5	95		
13.1	0.4	95	5		
16	0.4	95	5		

Table S3. Mass spectrometric parameters.

Options	Parameter
M/Z	50-1000
Ion Source Gas 1(psi)	50
Ion Source Gas 2(psi)	50
Curtain gas(psi)	30
Source temperature (℃)	500
IonSpray voltage floating (ESI+) (V)	5000
IonSpray voltage floating (ESI-) (V)	-4000
Interface heater	on
Declustering potential (V)	80
Collision energy (V)	5
MS/MS collision energy (V)	20-60(rolling)

Table S4. Biochemical and physiological test results of *P. megaterium* P-NA14 and *P. megaterium* D-HT207.

**Physiological and biochemical reactions**	**P-NA14**	**D-HT207**
Methyl red (M.R) test	+	+
Voges–Proskauer (V-P) test	−	−
Indole test	−	−

Hydrogen sulphide production test	+	+
Urea test	−	−
Catalase test	+	+
Starch hydrolysis test	+	+
Oxidase	+	+
Gelatin Liquefaction	+	+
Nitrate reduction	+	+
